# The diagonal branches and outcomes in patients with anterior ST- elevation myocardial infarction

**DOI:** 10.1186/s12872-020-01386-4

**Published:** 2020-03-04

**Authors:** Shuning Zhang, Xin Deng, Wenlong Yang, Liping Xia, Kang Yao, Hao Lu, Lei Ge, Li Shen, Aijun Sun, Yunzeng Zou, Juying Qian, Junbo Ge

**Affiliations:** 1 Department of cardiology, Zhongshan Hospital, Fudan University, Shanghai, China; 2 Shanghai Institute of Cardiovascular Diseases, Shanghai Cardiovascular Medical Center, Fudan University, Shanghai, China; 3 Department of cardiology, Shaoxing Shangyu People’s Hospital, Zhejiang, China; 4 Shanghai Institute of Cardiovascular Diseases, Shanghai Cardiovascular Medical Center, Fudan University, Shanghai, China; 5 Institutes of Biomedical Sciences, Fudan University, 1609Xietu Road, Shanghai, 200032 People’s Republic of China; 6 Institute of Pan-vascular Medicine, Fudan University, 1609Xietu Road, Shanghai, 200032 People’s Republic of China

**Keywords:** ST-segment elevation myocardial infarction, Primary percutaneous coronary intervention, Diagonal branches, Major adverse cardiac events

## Abstract

**Background:**

The management of diagonal branch (D) occlusion is still controversary. The association between the flow loss of D and the prognosis remains unclear. We aim to detect the impact of D flow on cardiac function and clinical outcomes in patients with anterior ST-segment elevation myocardial infarction (STEMI).

**Methods:**

Patients with anterior STEMI undergoing primary percutaneous coronary intervention (PCI) at our clinic between October 2015 and October 2018were reviewed. Anterior STEMI due to left anterior descending artery (LAD) occlusion with or without loss of the main D flow (TIMI grade 0–1 or 2–3) was enrolled in the analysis. The short- and long-term incidence of major adverse cardiac events (MACEs, a composite of all-cause death, target vessel revascularization and reinfarction) and left ventricular ejection fraction (LVEF) were analyzed.

**Results:**

A total of 392 patients (mean age of 63.9 years) with anterior STEMI treated with primary PCI was enrolled in the study. They were divided into two groups, loss (TIMI grade 0–1, *n* = 69) and no loss (TIMI grade2–3, *n* = 323) of D flow, before primary PCI. Compared with the group without loss of D flow, the group with loss of D flow showed a lower LVEF post PCI (41.0% vs. 48.8%, *p* = 0.003). Meanwhile, loss of D flow resulted in the higher in-hospital, one-month, and 18-month incidence of MACEs, especially in all-cause mortality (all *p* < 0.05). Landmark analysis further indicated that the significant differences in 18-month outcomes between the two groups mainly resulted from the differences during the hospitalization. In addition, multivariate Cox proportional hazards analysis found that D flow loss before primary PCI was independent factor predicting short- and long-term outcomes in patients with anterior STEMI.

**Conclusion:**

Loss of the main D flow in anterior STEMI patients was independently associated with the higher in-hospital incidences of MACEs and all-cause death as well as the lower LVEF.

## Introduction

ST-segment elevation myocardial infarction (STEMI),especially anterior STEMI, is the most severe type of coronary artery disease, with potential for substantial morbidity and mortality [1, 2]. In clinical practice, loss of diagonal branch (D) flow commonly occurs in patients with anterior STEMI undergoing primary percutaneous coronary intervention (PCI). Regardless of the presence of a left main artery lesion, loss of D flow is usually secondary to occlusion of the proximal segment of the left anterior descending artery (LAD) involving diagonal branches or isolated D occlusion. The LAD is the most common culprit vessel of anterior STEMI and its acute occlusion leads to greatly impaired cardiac function and unfavorable clinical outcomes [3, 4]. In contrast, isolated occlusion of the D is relatively rare for anterior STEMI and has little effect on cardiac function [5]; PCI of the D as a non-culprit vessel also fails to reduce the rate of adverse clinical outcomes [6, 7]. However, incidence of heart rupture is higher in STEMI patients involving the side branch than in those with the main branch occlusion alone [8]. In the scant literature available, the loss of D flow appears associated with more severe myocardial ischemia and the higher incidence of major adverse cardiac events (MACEs). However, it remains unclear whether the TIMI flow loss of the main D of LAD would lead to the worse clinical outcomes in patients with anterior STEMI. The present study aimed to detect the association between the baseline TIMI flow of D and cardiac function as well as outcomes of anterior STEMI subjects following primary PCI.

## Methods

### Study design

This single-center cohort study retrospectively enrolled consecutive patients with anterior STEMI undergoing primary PCI between Oct 2015 and Oct 2018 at our center. STEMI was defined as persistent chest pain with documented ST-segment elevation ≥1 mm in ≥2 contiguous leads or new left bundle branch block and elevated myocardial infarction markers. In all cases, culprit vessel was LAD with TIMI flow grade 0–1, with or without the involvement of the main D with diameter ≥ 2 mm, as evaluated by primary coronary angiography; and final TIMI flow of LAD and its D was grade 2–3 after primary PCI. Patients were excluded from analysis if they met any of the following criteria: presence of cancer, severe renal or hepatic dysfunction, age over 85 years, time from symptom onset to first medical contact ≥12 h, and unsuccessful interventional therapy. The patients included were divided into two groups based on TIMI flow grade at baseline in D by coronary angiography (TIMI grade 0–1; or TIMI grade2–3). In-hospital or post-discharge outcomes included all-cause mortality, target vessel revascularization (TVR), and myocardial reinfarction. The study protocol was approved by the ethics committee of Zhongshan Hospital, Fudan University.

### Primary percutaneous coronary intervention

All patients received dual antiplatelet therapy with aspirin (300 mg) and clopidogrel (300 mg) or ticagrelor (180 mg) before PCI. A statin and beta-blocker were prescribed to all the individuals without contraindications. A team of at least two experienced interventional cardiologists performed the PCI procedure. Choice of interventional strategies and devices was at the discretion of the operators. Procedural success was defined as the achievement of < 30%residual lumen stenosis with Thrombolysis in Myocardial Infarction (TIMI) flow grade 2–3 in the target vessel. All cases received standard perioperative hospital care.

### Data collection

Sociodemographic and laboratory data were extracted from the electronic medical management system at our hospital. Leucocyte count, and levels of serum cardiac troponin T (cTNT), creatine kinase myocardial band (CK-MB), N-terminal pro B type natriuretic peptide (NT-proBNP), serum creatinine (Scr) and C-reactive protein (CRP) were measured in the blood sample drawn on admission. The serum lipid profile was tested on the next morning. Serum levels of cTNT, CK-MB, and NT-proBNP also were assessed at days 1, 3, and 7 after PCI, and their peak values were collected. All patients underwent echocardiographic assessment within 24 h post primary PCI and the findings were recorded. The angiographic findings were evaluated by two experienced interventional cardiologists. In-hospital clinical outcomes were collected by reviewing medical records. Post-discharge outcomes were assessed by telephone or clinical interview. Nearly all the patients (362 out of 368 patients) completed the outpatient visit once at least after PCI and followed the medication of the doctor during the follow-up.

### Statistical analysis

Continuous variables are presented as mean ± standard deviation (SD) when normally distributed as per the Kolmogorov-Smirnov test or otherwise as median (interquartile range). Categorical variables are expressed as number (%). Continuous variables were compared using student’s *t* test or Mann-Whitney U test. Categorical variables were compared using the Chi-square test (or Fisher exact test). Univariate analysis was used to evaluate the effect of different variables on MACE. Suspected variables or variables with *p* < 0.1 in univariate analysis were included into the multivariate Cox proportional hazards model. Sensitivity analysis was performed to determine whether the association with outcomes differed by using three different Cox multivariable models. Collinearity diagnostics was completed before the multivariable analysis, whose results were presented as hazard ratios (HR) and 95% confidence intervals (95% CI). Survival curves were analyzed using Kaplan-Meier estimation and were compared using the log-rank test. A landmark analysis was performed to assess the long-term mortality in patients who survived at discharge. *P* value < 0.05 was considered statistically significant. Data analyses were conducted using SPSS 25.0 software (IBM SPSS Statistics, USA).

## Results

### Baseline demographics and clinical characteristics

**Fig. 1:**
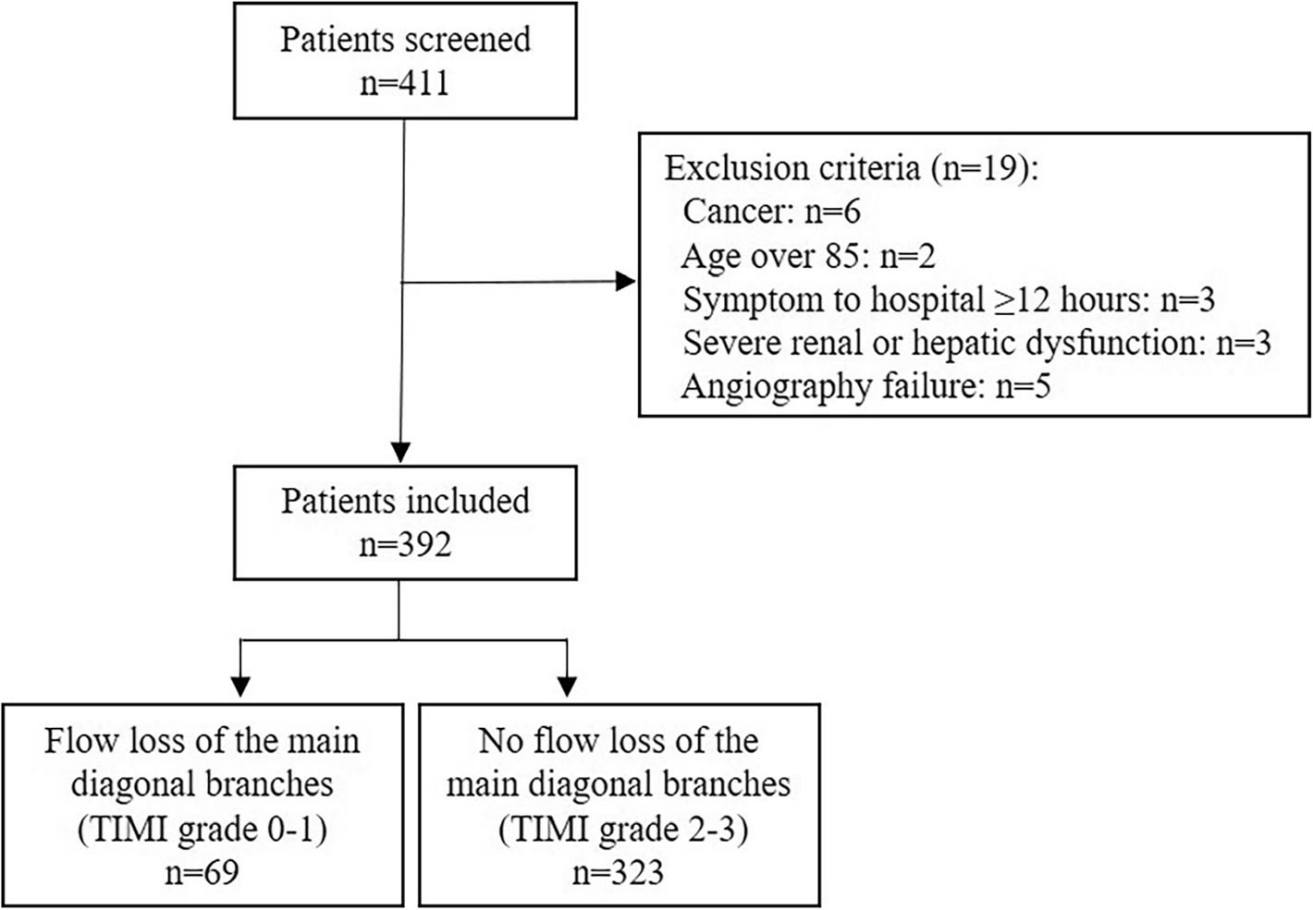
Patient selection flow diagram

In the present study, 411patients with anterior STEMI undergoing primary PCI were initially reviewed, and 19 patients were excluded (Fig. 1). A total of 392anterior STEMI patients were included in this analysis. Mean age of patients was 63.9 years, and 82.7% were male. Time from symptom onset to first medical contact did not exceed 3 h in 22.4% of patients. Left ventricular ejection fraction (LVEF) within 24 h after primary PCI remained 47.4 ± 13.2%, and patients with Killip class> 2accounted for 4.1% (16 patients) (Table 1). Among the enrolled 392 subjects, 53.3% presented multivessel disease, 9.9% was concomitant with coronary chronic total occlusion lesion, and 93.1% underwent stent implantation during primary PCI (Table 2).

**Table 1:** Demographic and laboratory characteristics at baseline

Variables	Total (*n* = 392)	Flow of the main diagonal branches of LAD		*p* Value
		loss (TIMI 0–1) (*n* = 69)	no loss (TIMI 2–3) (*n* = 323)	
Age, years, mean ± SD	63.9 ± 11.6	64.7 ± 12.2	63.8 ± 11.4	0.60
Female, n(%)	68(17.3)	14(20.3)	54(16.7)	0.48
Diabetes, n(%)	103(26.3)	24(34.8)	79(24.5)	0.08
Hypertension, n(%)	232(59.2)	38(55.1)	194(60.1)	0.44
Smoking history, n(%)	164(41.8)	22(31.9)	142(44)	0.07
Stroke, n(%)	26(6.6)	6(8.7)	20(6.2)	0.45
Family history of CAD, n(%)	8(2)	0	8(2.5)	0.36
Previous CAD, n(%)	26(6.6)	6(8.7)	20(6.2)	0.43
Valve calcification, n(%)	86(21.9)	13(18.8)	73(22.6)	0.49
Time from symptom onset to hospital, hours	6(4–10)	6(4–9)	6(4–10)	0.35
0-3 h, n(%)	88(22.4)	15(21.7)	73(22.6)	0.42
3-6 h, n(%)	117(29.8)	25(36.2)	92(28.5)	
6-12 h, n(%)	187(47.7)	29(42)	158(48.9)	
LVEF, %, mean ± SD	47.4 ± 13.2	41 ± 18.3	48.8 ± 11.4	0.003
Killip class> 2, n(%)	16(4.1)	8(11.6)	8(2.5)	0.001
SBP, mmHg, mean ± SD	123 ± 31.6	109.0 ± 34.3	126.1 ± 30.2	0.36
TC, mmol/L, mean ± SD	4.4 ± 1.1	4.6 ± 1.3	4.3 ± 0.9	0.09
LDL-C, mmol/L	2.6(2–3.2)	2.5(1.7–3.3)	2.5(1.9–3.2)	0.89
CRP, mg/L	6.7(3–12.1)	4.2(1–10.2)	6.6(3–10.8)	0.23
Scr, μmol/L	68.5(57–82.8)	73.5(63.8–86)	71(60–87)	0.71
cTNT, ng/ml	0.2(0.05–0.75)	0.11(0.04–0.64)	0.26(0.06–0.78)	0.08
CKMB, U/L	27(14–63)	21(14.8–104)	30.5(16–69.3)	0.46
NT-proBNP, pg/ml	227.6(59.4–922.9)	126.1(49.4–429.5)	236.4(57.7–1142.3)	0.16
Medications during hospital				
Aspirin, n(%)	363(92.6)	64(92.8)	299(92.6)	0.96
Clopidogrel, n(%)	247(63)	41(59.4)	206(63.8)	0.50
Ticagrelor, n(%)	145(37)	28(40.6)	117(36.2)	0.49
GPIIb/IIIa receptor antagonists, n(%)	248(63.3)	44(63.8)	204(63.2)	0.91
AECI/ARB, n(%)	381(97.2)	67(97.1)	314(97.2)	0.98
β-receptor blocker, n(%)	387(98.7)	68(98.6)	319(98.8)	0.98
Statin, n(%)	392(100)	69(100)	323(100)	0.99
Hospital stay, days, mean ± SD	8.1 ± 3.8	8.9 ± 4	7.9 ± 3.8	0.07

*SD* Standard deviation, *LAD*, Left anterior descending artery, *CAD* Coronary artery diseasem, *SBP* Systolic blood pressure, *TC* Total cholesterol, *LDL* Low density lipoprotein, *CRP* C–reactive protein, *Scr* Serum creatinine, *cTNT* Cardiac troponin T, *CK-MB* Creatine Kinase MB; *LVEF* Left ventricular ejection fraction, *NT-proBNP* N-terminal pro B-type natriuretic peptide, *ACEI/ARB* Angiotensin converting enzyme inhibition/angiotensin II receptor blockers

**Table 2:** Angiographic Characteristics

Variables	Total (*n* = 392)	Flow of the main diagonal branches of LAD		*p* Value
		loss (TIMI 0–1) (*n* = 69)	no loss (TIMI2–3) (*n* = 323)	
Complicated chronic total occlusion, n(%)	39(9.9)	7(10.1)	32(9.9)	0.95
Complicated multi-vessel disease, n(%)	209(53.3)	33(47.8)	176(54.5)	0.31
Complicated LM lesion, n(%)	24(6.1)	11(15.9)	13(4)	0.001
Visully thrombus, n(%)	187(47.7)	49(83.1)	138(54.3)	0.001
Intervention for culprit vessel				
1st generation DES, n(%)	202(51.5)	31(44.9)	171(52.9)	0.42
2nd generation DES, n(%)	163(41.6)	32(46.4)	131(40.6)	0.82
No stent implantation, n(%)	27(6.9)	6(8.7)	21(6.5)	0.51
Stent diameter, mm	3.1 ± 0.4	3.3 ± 0.4	3.1 ± 0.3	0.001
Stent length, mm	27.5 ± 6.3	24.9 ± 6.3	28 ± 6.1	0.001

*LAD* Left anterior descending artery, *LM* Left main artery *DES* Drug-eluting stent

Of 392 patients, 17.6% (69 patients) presented acute occlusion of LAD concomitant with flow loss of its main D and 82.4% without D flow loss. Compared with patients without flow loss, those with D flow loss had a greater Killip class, a lower LVEF, and a higher proportion of LM lesions. Symptom to hospital time, age, sex, medical history, medication at discharge, laboratory, and angiographic characteristics were similarly distributed between groups.

### Clinical outcomes

During the mean follow-up of 18 months, a total of 38 (9.7%) patients suffered from MACEs, including 24 all-cause death, 3 TVR, and 11 non-fatal myocardial reinfarction. Among 19 in-hospital deaths, 13 died from myocardial infarction complications, heart failure, cardiac shock and malignant arrhythmia, 6 died from severe systemic infection, respiratory failure and multiple organ dysfunction syndrome. The one-month and 18-month MACEs rate was 14.5 and 17.4% in the group with D flow loss, and 4 and 8%in the group without D flow loss(p [one-month] =0.003; p [18-month] =0.017, Table 3). Moreover, there were also significant intergroup differences in all-cause mortality and cardiac mortality (p [one-month] = 0.001; p [18-month] =0.001). Other outcomes such as TVR, myocardial reinfarction, and length of hospital stay were similarly distributed between the two groups. In additions, the landmark analysis showed that incidences of MACEs and all-cause mortality did not indicate marked intergroup differences both in the intervals of leaving hospital to 18-month post discharge (MACEs: 3.4% vs. 5.4%, *p* = 0.74; all-cause mortality: 3.4% vs. 1%, *p* = 0.38, respectively), and in the intervals of one-month to 18-month (3.4% vs. 4.2%, *p* = 0.77; 3.4% vs. 1%, *P* = 0.21, respectively).

**Table 3:** Short- and long-term MACEs

Variables	Total (*n* = 392)	Flow of the main diagonal branches of LAD		*p* Value
		loss (TIMI 0–1) (*n* = 69)	no loss (TIMI2–3) (*n* = 323)	
In-hospital MACEs	19(4.8)	10(14.5)	9(2.8)	0.001
All-cause mortality, n(%)	19(4.8)	10(14.5)	9(2.8)	0.001
Cardiac mortality, n(%)	13(3.3)	10(14.5)	3(0.9)	0.001
TVR, n(%)	0	0	0	/
Recurrent myocardial infarction, n(%)	0	0	0	/
1-month MACEs	23(5.9)	10(14.5)	13(4)	0.003
All-cause mortality, n(%)	19(4.8)	10(14.5)	9(2.8)	0.001
Cardiac mortality, n(%)	13(3.3)	10(14.5)	3(0.9)	0.001
TVR, n(%)	1(0.3)	0	1(0.3)	1
Recurrent myocardial infarction, n(%)	3(0.8)	0	3(0.9)	1
18-month MACEs	38(9.7)	12(17.4)	26(8)	0.02
All-cause mortality, n(%)	24(6.1)	12(17.4)	12(3.7)	0.001
Cardiac mortality, n(%)	16(4.1)	10(14.5)	6(1.9)	0.001
TVR, n(%)	3(0.8)	0	3(0.9)	1
Recurrent myocardial infarction, n(%)	11(2.8)	0	11(3.4)	0.23
18-month MACEs (landmark analysis^a^)	15(4.1)	2(3.4)	13(4.2)	0.77
All-cause mortality, n(%)	5(1.4)	2(3.4)	3(0.9)	0.21
Cardiac mortality, n(%)	3(0.8)	0	3(0.9)	1
TVR, n(%)	2(0.5)	0	2(0.7)	1
Recurrent myocardial infarction, n(%)	8(2.2)	0	8(2.6)	0.37
18-month MACEs (landmark analysis^b^)	19(5.1)	2(3.4)	17(5.4)	0.74
All-cause mortality, n(%)	5(1.3)	2(3.4)	3(0.9)	0.38
Cardiac mortality, n(%)	3(0.8)	0	3(0.9)	1
TVR, n(%)	3(0.8)	0	3(0.9)	1
Recurrent myocardial infarction, n(%)	11(2.9)	0	11(3.5)	0.23

^a^analysis starting from 1 month to 18 months. ^b^analysis starting from the time of discharged the hospital to 18 months. *LAD* Left anterior descending artery, *MACEs* Major adverse cardiac events, *TVR* Target vessel revascularization

In multivariate Cox proportional hazards analysis, TIMI flow grade 0–1of D was significantly associated with one-month MACEs (Model 1: HR 2.96, 95% CI 1.27–6.94, *p* = 0.01;Model 2: HR 2.99,95% CI1.26–7.08, *p* = 0.01). Furthermore, D flow loss was also an independent risk factor for 18-month MACEs (Model 1: HR 2.52, 95% CI1.23–5.17, *p* = 0.01;Model 2: HR 2.43,95% CI1.19–4.95, *p* = 0.02) (Table 4).

**Table 4:** Univariate and multivariate analysis of MACEs

	One-month MACE			Long-term MACE		
	HR	95% CI	*p* Value	HR	95% CI	*p* Value
Univariable analysis						
Age, years	1.07	1.03–1.11	0.001	1.05	1.02–1.09	0.001
Male, gender	0.6	0.24–1.51	0.27	0.64	0.3–1.36	0.25
previous MI	1.82	0.25–13.48	0.56	2.32	0.56–9.65	0.25
Killip class> 2	11.1	4.37–28.22	0.001	9.1	4.16–19.87	0.001
Symptom to hospital time ≤ 3 h	0.51	0.15–1.72	0.28	0.65	0.27–1.55	0.33
Scr	1.003	1–1.005	0.06	1.005	1–1.01	0.001
Preserved LVEF	0.15	0.04–0.65	0.01	0.31	0.13–0.74	0.01
cTNT	0.97	0.78–1.22	0.81	1.08	0.99–1.18	0.09
Chronic total occlusion	3.44	1.36–8.74	0.01	3.15	1.49–6.66	0.003
LM lesion	2.49	0.74–8.37	0.14	2.12	0.75–5.99	0.16
TIMI grade0–1	3.88	1.70–8.86	0.001	2.4	1.21–4.77	0.01
Multivariable analysis						
Model 1						
TIMI grade 0–1	2.96	1.27–6.94	0.01	2.52	1.23–5.17	0.01
Age	1.06	1.02–1.11	0.003	1.04	1.01–1.08	0.01
Killip class> 2	6.23	2.35–16.52	0.001	5.04	2.19–11.61	0.001
Model 2						
TIMI grade 0–1	2.99	1.26–7.08	0.01	2.43	1.19–4.95	0.02
Age	1.05	1.02–1.09	0.01	1.04	1.01–1.07	0.01
Killip class> 2	4.76	1.8–12.61	0.002	4.73	2.05–10.89	0.001
Preserved LVEF	0.24	0.05–1.03	0.06	0.41	0.17–1.01	0.05
Chronic total occlusion	3.26	1.26–8.43	0.02	3.48	1.6–7.56	0.002
Scr	1.003	1–1.007	0.09	1.006	1.004–1.007	0.001

*LAD* Left anterior descending artery, *MACEs* Major adverse cardiac events, *MI* Myocardial infarction, *Scr* Serum creatinine, *cTNT* Cardiac troponin T, *LVEF* Left ventricular ejection fraction, *LM* Left main artery, *CTO* Chronic total occlusion

**Fig. 2:**
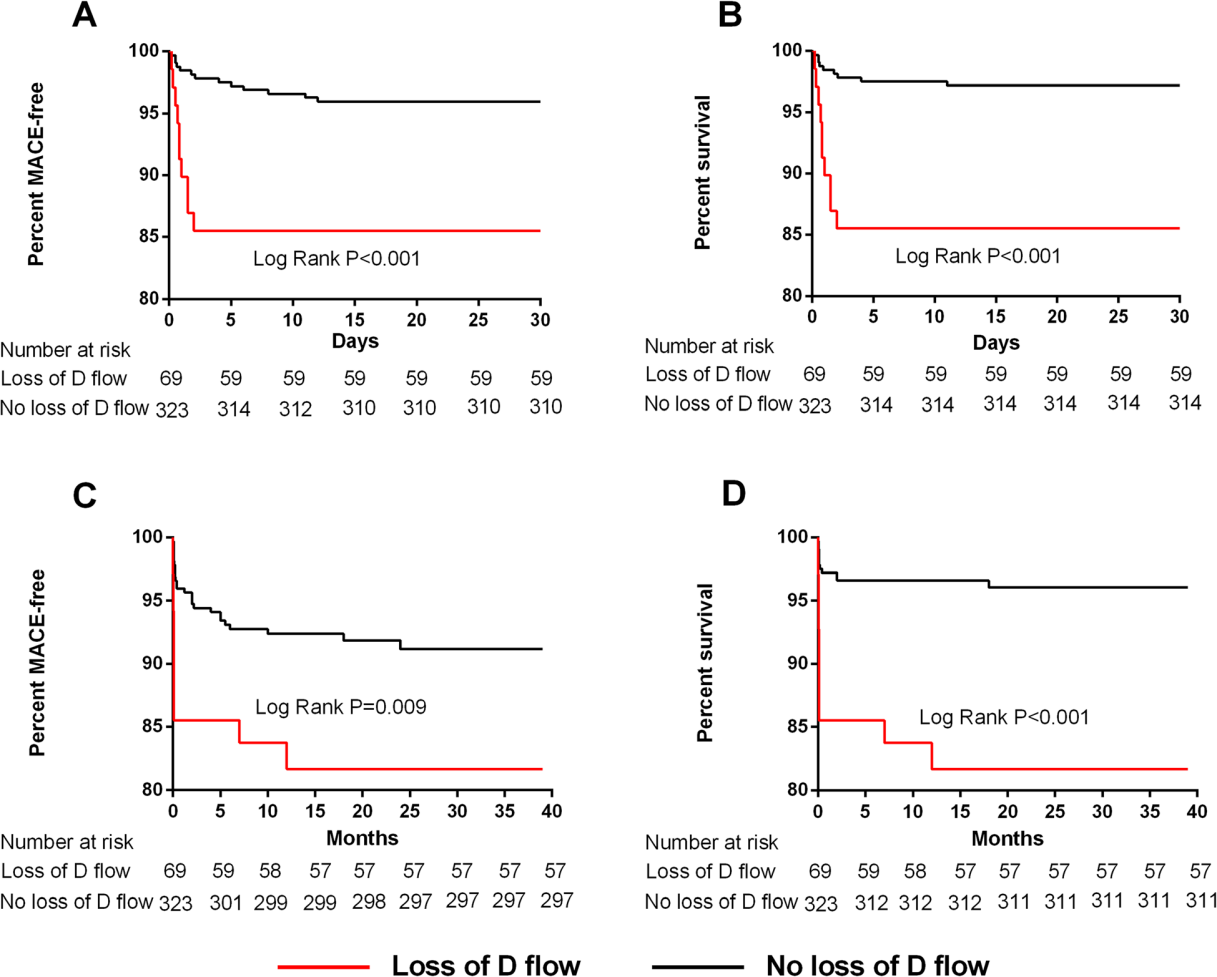
Kaplan-Meier survival analysis of MACE-free (**a**) rate curve at one month and 18 month (**c**) and survival rate curve at one month (**b**) and 18 month (**d**). MACE, major adverse cardiac event

Kaplan-Meier survival curves showed that there were significant differences in MACEs at 1 month (Log rank *p* < 0.001, Fig**.** 2-A) and 18 months (Log rank *p* = 0.009, Fig**.**2-C) between the two groups, which was also the case for Kaplan-Meier survival curves for all-cause mortality (one-month: Log rank *p* < 0.001, Fig**.**2-B; and 18-month: Log rank *P* < 0.001, Fig**.**2-D). The group without D flow loss had a higher survival rate and a lower MACEs rate not only at 1 month but also at 18 months post STEMI (Fig. 2).

## Discussion

This retrospective study showed that loss of the main D flow before primary PCI of anterior STEMI was associated with worse one-month and long-term adverse clinical outcomes, even after the active revascularization. It suggested need to pay more attention to the treatment and follow-up of these patients.

In general, LAD supplies 40% of the myocardium in the left ventricle, including the anterior ventricular septum and the anterior wall of the left ventricle [9]. Prognosis of LAD occlusion is related to the location of the lesion [3, 10]. The main D, usually referring to the first D, is the most important branch of the LAD supplying blood to the anterior and anterolateral wall of the left ventricle. Because the D has different diameters, lengths, and range distributions in different populations, the degree of influence of occlusion on cardiac function is different when the D is occluded. Generally, the number of D ranges from 2 to 9; a D with diameter larger than 2 mm is regarded as the main supply branch of the LAD. The previously published researches on the D were mostly about the bifurcation lesion of LAD/D, with scarce study of the D itself [11].

The proximal LAD was defined as the segment spanning from the ostium of the LAD to that of the first main branch (usually referring to the significant diagonal branch) [12]. Generally, both proximal LAD and D occlusion can cause loss of D flow. Loss of D flow might be associated with poor prognosis of patents with STEMI. However, the clinical significance of the two occlusion scenarios is quite different, with the former being most common, of greater severity, and with worse prognosis [11, 13, 14], while isolated D occlusion is relatively rare and appears mainly inconsequential [15]. Therefore, distinction between the two scenarios is of clinical relevance. Electrocardiographic findings may provide clues [16, 17], and coronary angiography can basically distinguish between the two scenarios. Regarding therapy, proximal LAD occlusion compromising the main D flow should undoubtedly be treated by revascularization as soon as possible. In contrast, the treatment of isolated D occlusion is currently a matter of debate [7, 18]. It is not possible to generalize about the need to perform further revascularization for the D after intervention of the target vessel, because different individuals have different D blood flow conditions and the target lesions are different. However, published studies showed that complete revascularization is a reasonable strategy to improve outcomes in patients with STEMI [19, 20]. In clinical practice, we are inclined to perform PCI for the side branch with diameter ≥ 2 mm. The benefit and risk should be comprehensively weighed according to various factors like age, lesion severity, renal function and procedure duration. The decision that whether treating side branch are supposed to be made carefully.

Isolated D occlusion has been reported to have a lesser effect on cardiac function [5]. In our study, the proportion of patients with normal LVEF in the group with D flow loss was lower than that in the group without D flow loss. Therefore, the group with D flow loss had a larger myocardial infarct size, lower cardiac function and a higher Killip class grade. Regarding the potential cause of worse clinical outcomes, we speculated that it could be explained partly by the proximal LAD occlusion. Almost all the patients with D flow loss in our study presented with LAD lesions involving the D. However, we believed that for the proximal LAD occlusion patients, those with D flow loss were still more likely to have a larger myocardial infarct size and worse outcomes compared with those without D flow loss. The cardiac MRI test could accurately measure infarct size and verify our viewpoint. Regrettably, we failed to obtain the MRI data in this retrospective study. As the loss of the main D often results from proximal LAD lesions, the average diameter of LAD and the stent implanted in the group with D flow loss might be larger, which was verified in the present study.

Due to the relatively large sample size difference between the two groups, we performed multivariate analysis using two COX models, which presented the stable and close results. The sensitivity analysis further demonstrated that the loss of the D was independently associated with clinical adverse events at 1 month and 18-month follow-up. In the Kaplan-Meier survival curves, clinical adverse events, particularly all-cause mortality, occurred in the group with D flow loss, mainly within 30 days after the intervention, underscoring the relevance of appropriate early postprocedural care. The landmark analysis further showed that the differences in the incidences of MACEs and all-cause death between the two groups mainly resulted from in-hospital events. However, the incidences of MACEs and all-cause death after discharge were similar between groups.

## Limitations

The present study has several limitations including those inherent to its retrospective, single center design. First, LVEF within the first 24 h of STEMI was unable to reflect the exact cardiac function and we did not follow up LVEF. Moreover, infarct size was not estimated on admission. Besides, there might be recallbias about the drug usage during follow-up. As thus, large, prospective, randomized clinical trials are required to confirm our findings in the present study.

## Conclusions

In summary, these findings based on the observational study demonstrated that patients with loss of the main D flow following anterior STEMI might be associated with the lower LVEF and the higher incidence of MACEs and all-cause mortality compared to those with the preserved D flow. The involvement of the main D due to LAD occlusion was an independent factor impacting clinical prognosis of anterior STEMI. Overall, loss of the main D flow following anterior STEMI suggested a relatively worse clinical prognosis, and the need for more active observation and follow-up, especially during the early stage of STEMI post primary PCI.

## Abbreviations

CK-MB: Creatine kinase myocardial band
CRP: C-reactive protein
cTNT: Cardiac troponin T
D: Main diagonal branch
LAD: Left anterior descending artery
LVEF: Left ventricular ejection fraction
MACE: Major adverse cardiac event
NT-proBNP: N-terminal pro B type natriuretic peptide
PCI: Percutaneous coronary intervention
Scr: Serum creatinine
STEMI: ST-segment elevation myocardial infarction
TIMI: Thrombolysis in myocardial infarction
TVR: Target vessel revascularization
